# Supplementary material for: Photocatalytic oxidation of methane over silver decorated zinc oxide nanocatalysts
Source: Nat Commun. 2016 Jul 20;7:12273. doi: 10.1038/ncomms12273 (PMC4961799; doi:10.1038/ncomms12273)
Supplement: Supplementary Information — Supplementary Figures 1-12 and Supplementary Note 1. [file ncomms12273-s1.pdf]

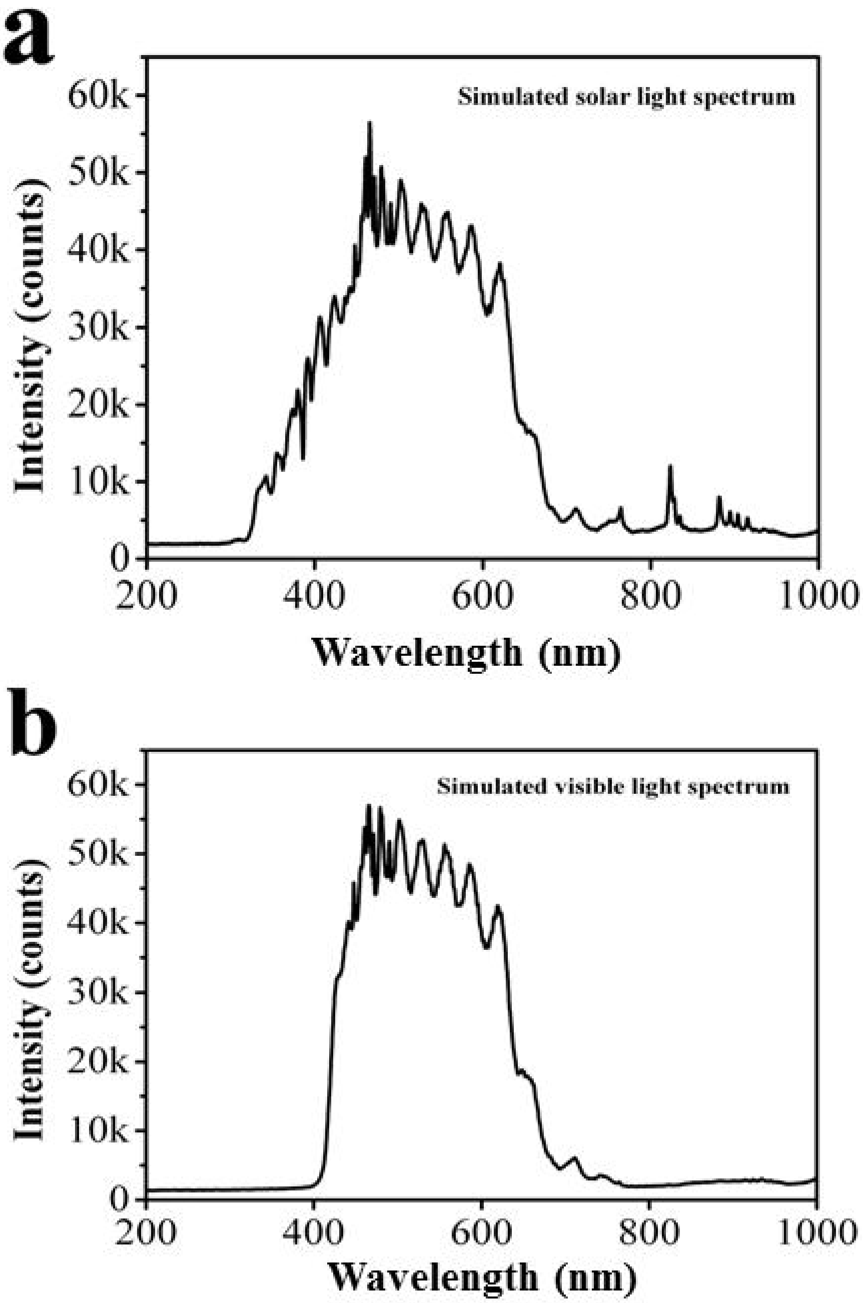

Supplementary Figure 1: The spectrum of simulated solar light. a, UV-Vis spectrum; **b**, visible light spectrum.

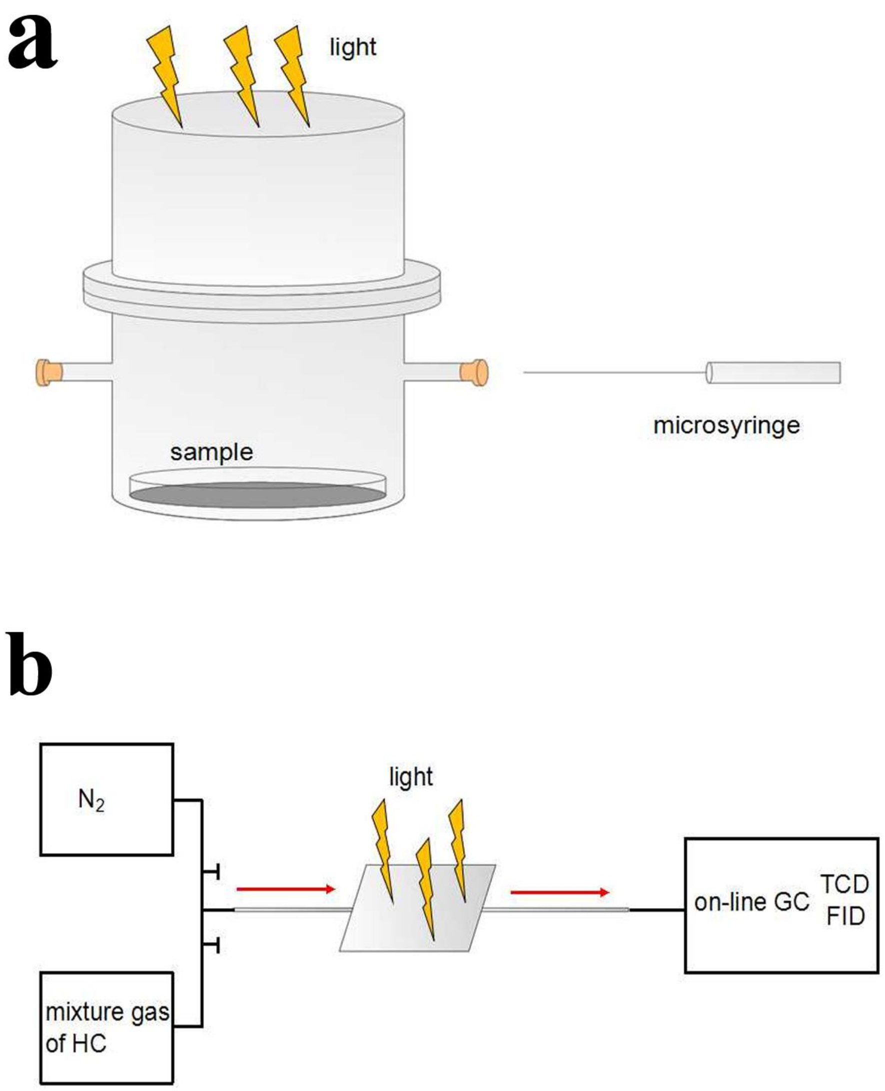

Supplementary Figure 2: The schematic diagram of photocatalytic instruments. **a**, fixed-bed mode; **b**, flow-bed mode. For operation details please see the section of Photocatalytic experiments in Methods following the manuscript text.

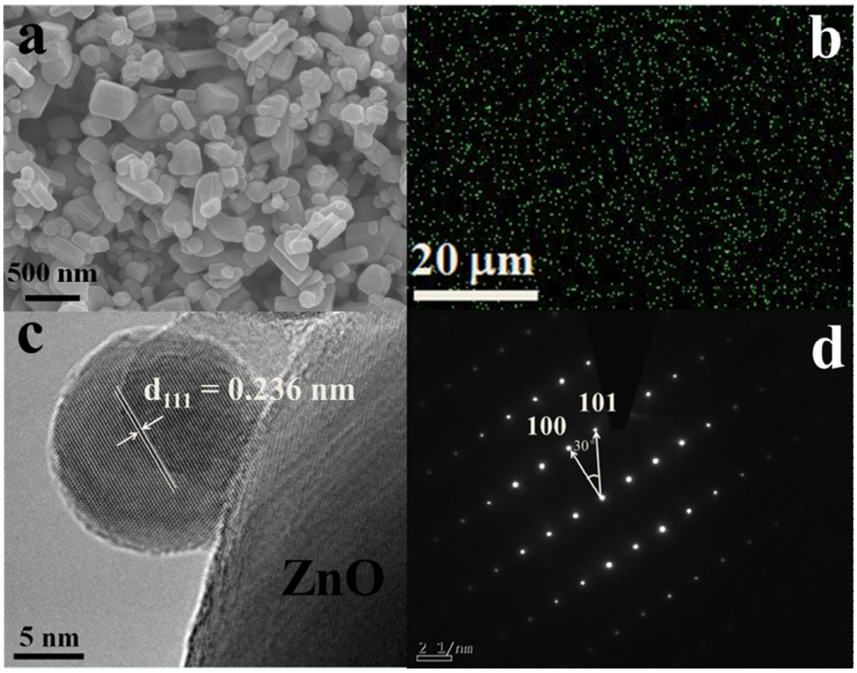

Supplementary Figure 3: Characterization of the commencial ZnO that was decoration with 0.5wt% Ag. a, SEM image; **b**, Elemental mapping of Ag (No obvious Ag aggregation was detected. Note the weak signal of Ag in the elemental mapping might come from the background noise whilst the silver content is low); **c**, HRTEM image distinguishes Ag and ZnO; **d**, Selected-area electron diffraction pattern of ZnO.

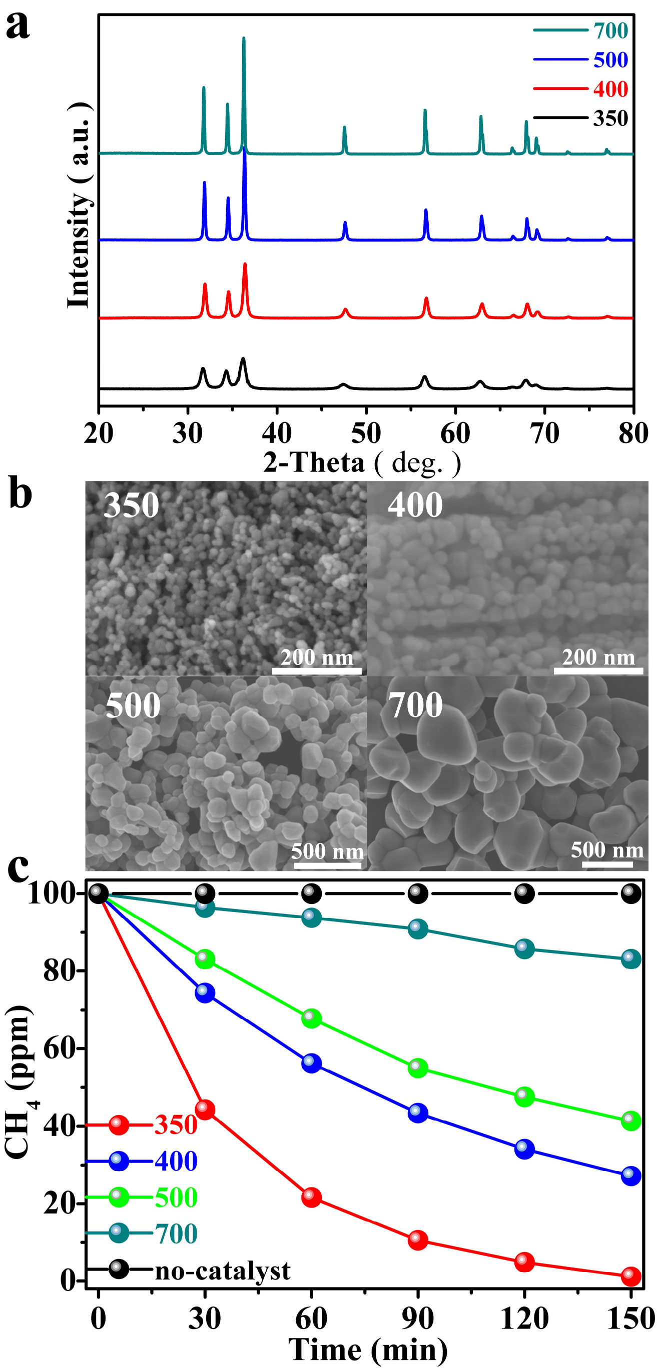

Supplementary Figure 4: Size effect of ZnO on photocatalytic methane oxidation. **a**, Room temperature XRD patterns of ZnO powders prepared at various calcination temperatures (^{o}C). **b**, SEM images of ZnO powders prepared at various calcination temperatures (^{o}C). The particles sizes are ~20, 25-40, 100-150, and 300-500 nm, respectively. **c**, Time course of methane photooxidation with and without the ZnO samples that were prepared at various calcination temperatures (^{o}C) Test mode: the fixed-bed with full arc illumination.

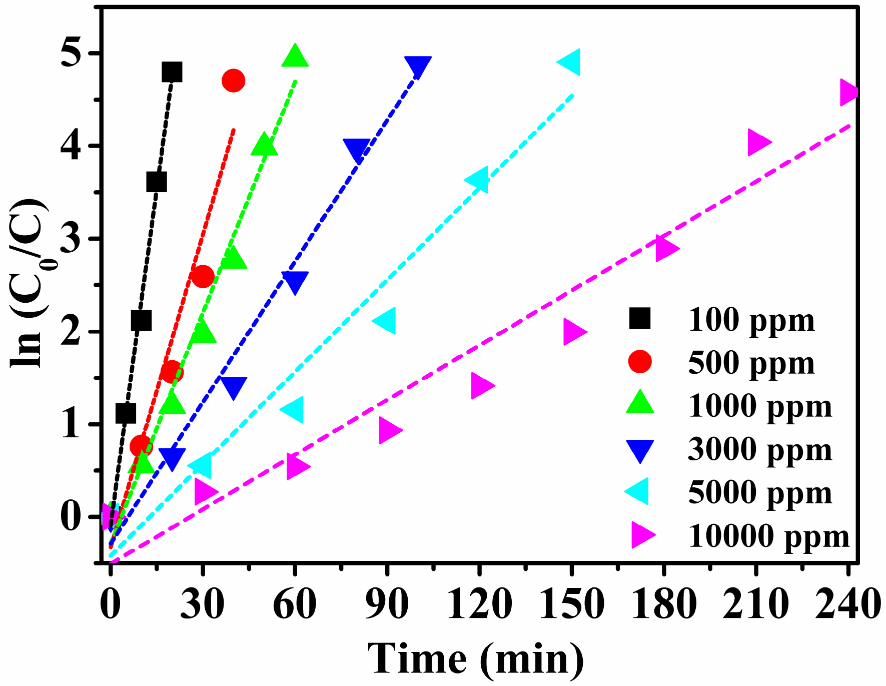

Supplementary Figure 5: Pseudo-first-order reaction kinetics plots of photocatalytic methane oxidation over the 0.1-Ag-ZnO samples with various CH_4 concentrations. The rate constants *k* deduced are 0.242, 0.112, 0.083, 0.051, 0.033 and 0.020 min^{-1}, respectively, with increasing the methane concentration from 100 to 10000 ppm.

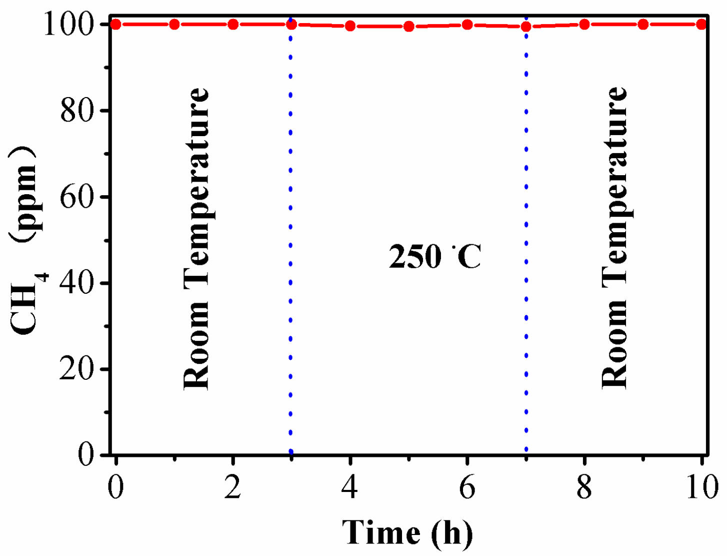

Supplementary Figure 6: Examination of the impact of temperature on methane oxidation over the 0.1-Ag-ZnO nanocatalyst in the flow-bed mode with a gas flow rate of 10 mL/min and without light illumination.

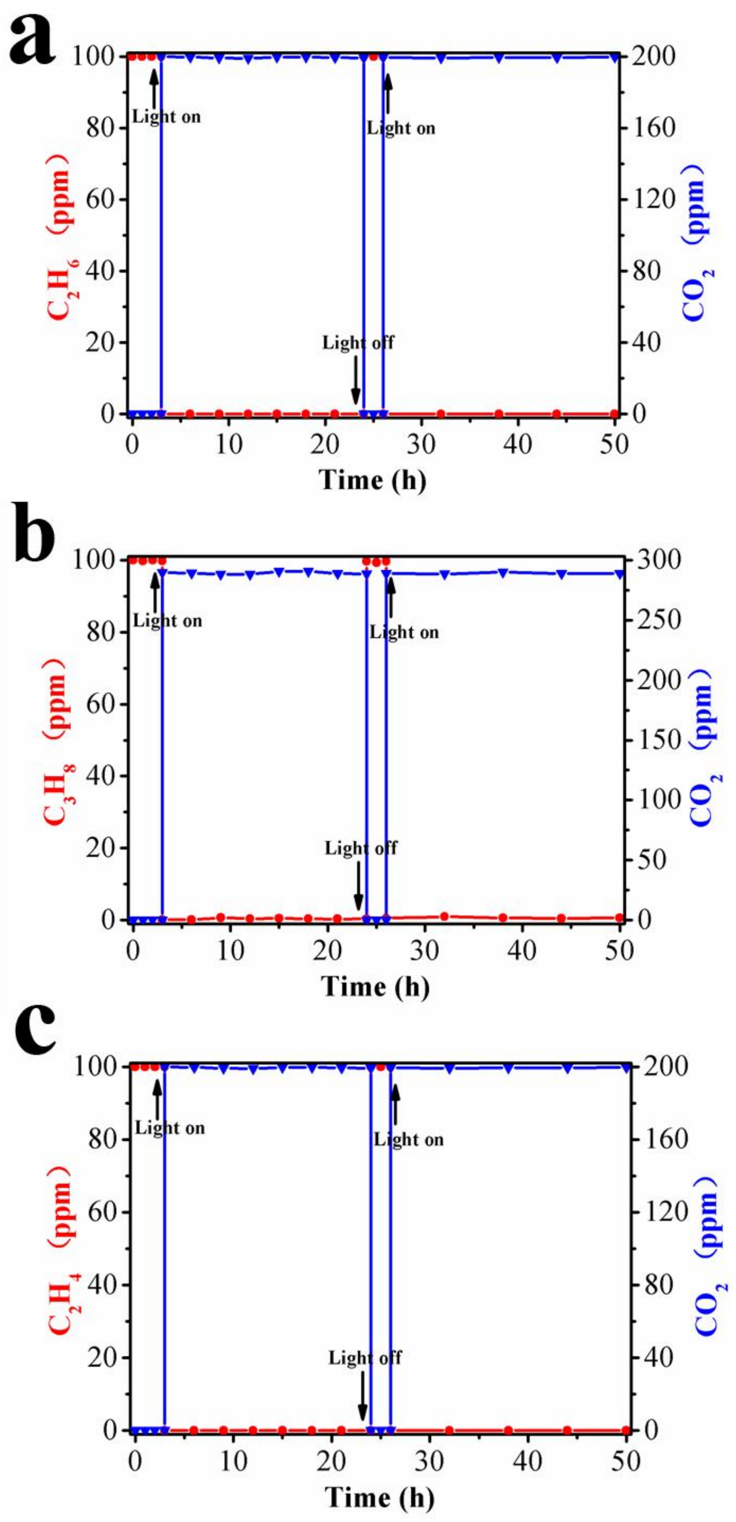

Supplementary Figure 7: Time courses of photocatalytic oxidation of various hydrocarbons under simulated sunlight over the 0.1-Ag-ZnO nanocatalysts in the flow-bed mode with a gas flow rate of 20 mL/min: **a**, C_2H_6; **b**, C_3H_8; **c**, C_2H_4.

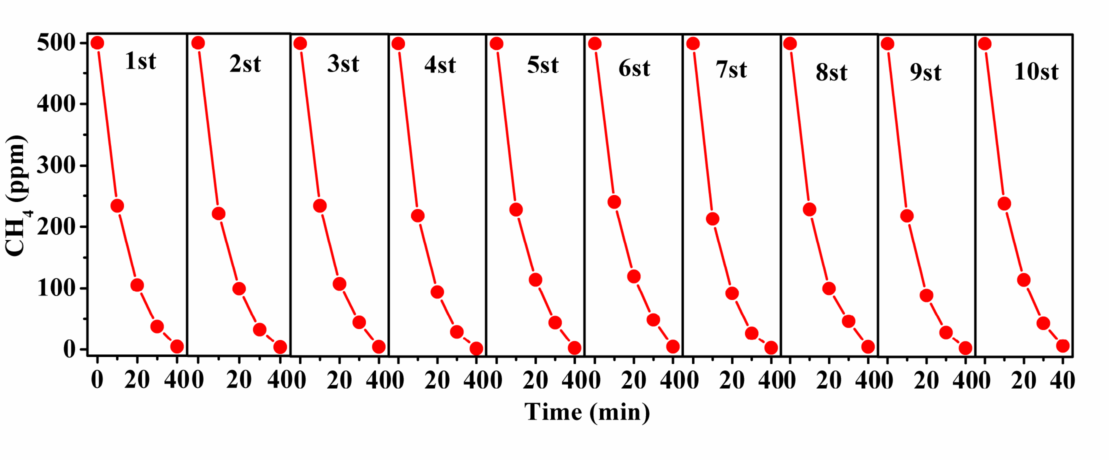

Supplementary Figure 8: Recycled test of photocatalytic activity of the 0.1-Ag-ZnO toward methane (500 ppm) oxidation in a fixed-bed reactor under simulated solar light illumination.

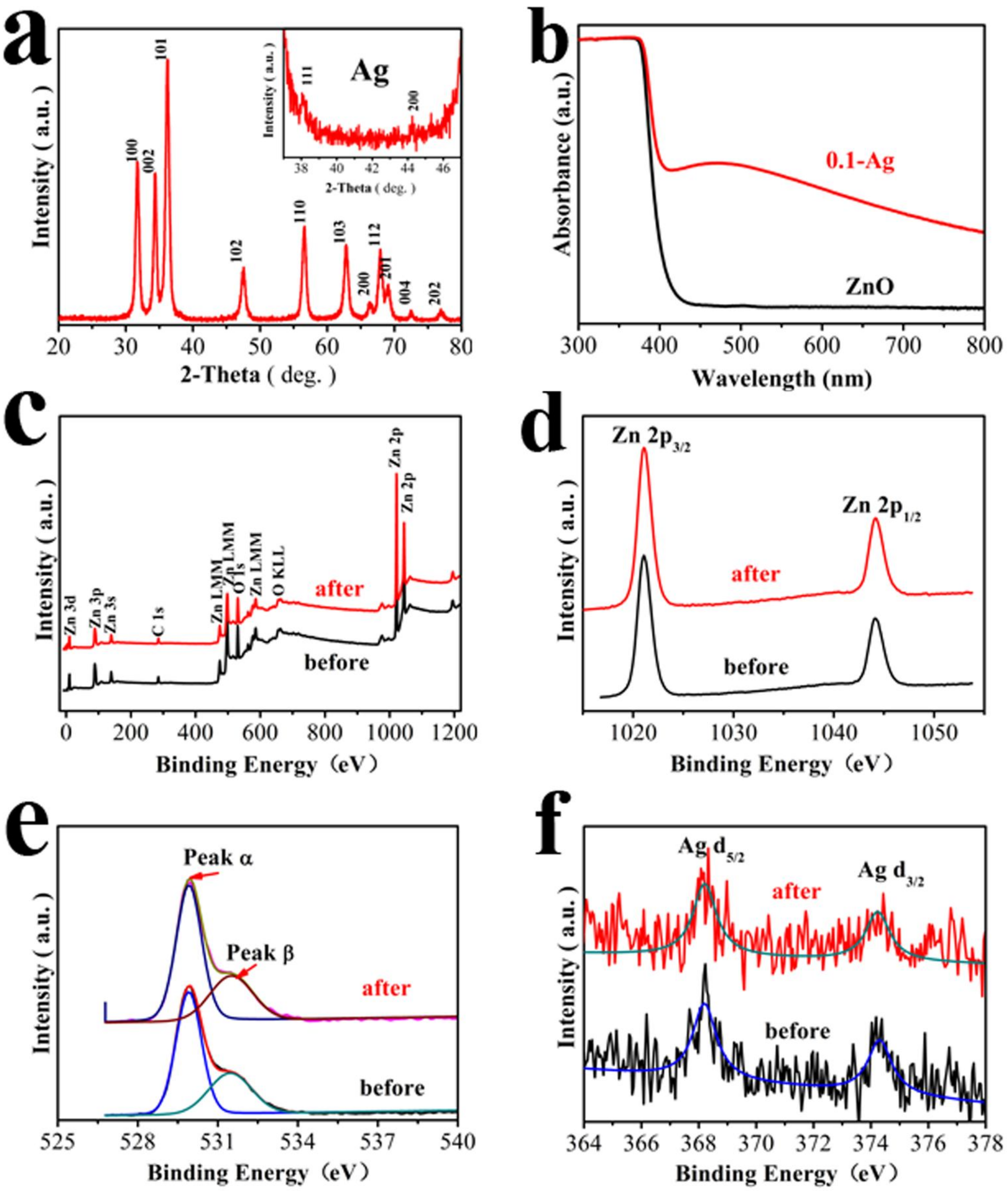

Supplementary Figure 9: Physical characterization of the 0.1-Ag-ZnO nanocatalysts after the photocatalytic reactions. **a**, XRD patterns; **b**, UV-Vis spectra; **c**, Full XPS spectra; **d**, XPS spectra of Zn $2p_{3/2}$ and Zn $2p_{1/2}$; **e**, XPS spectra of O 1s; **f**, XPS spectra of Ag $3d_{5/2}$ and Ag $3d_{3/2}$.

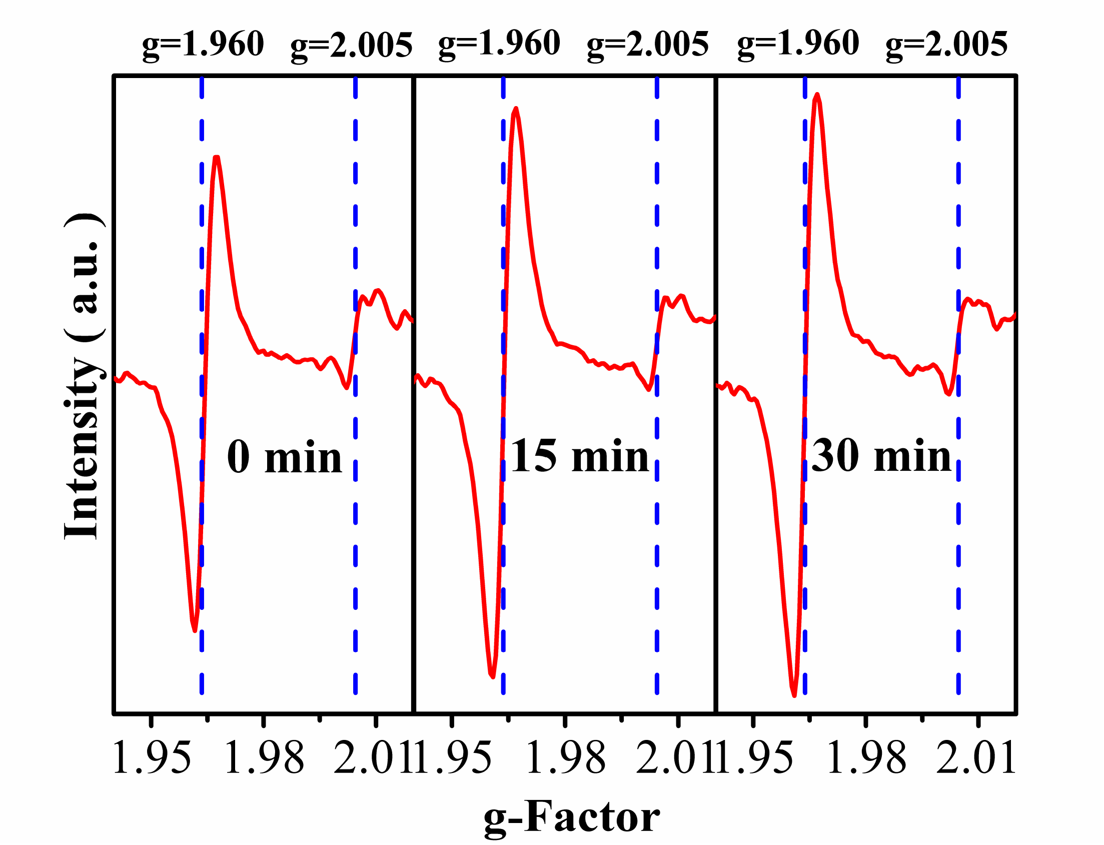

Supplementary Figure 10: Evolution of EPR signals when keep illuminating the 0.1-Ag-ZnO sample in the atmosphere containing methane and oxygen.

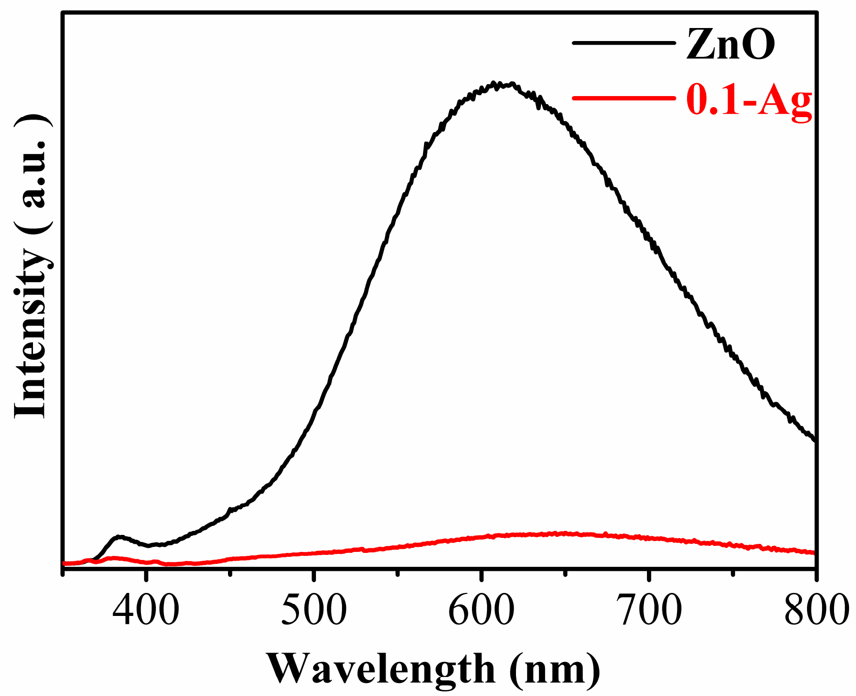

Supplementary Figure 11: Photoluminescence (PL) spectra of the nano-particulate ZnO and its Ag decorated counterpart.

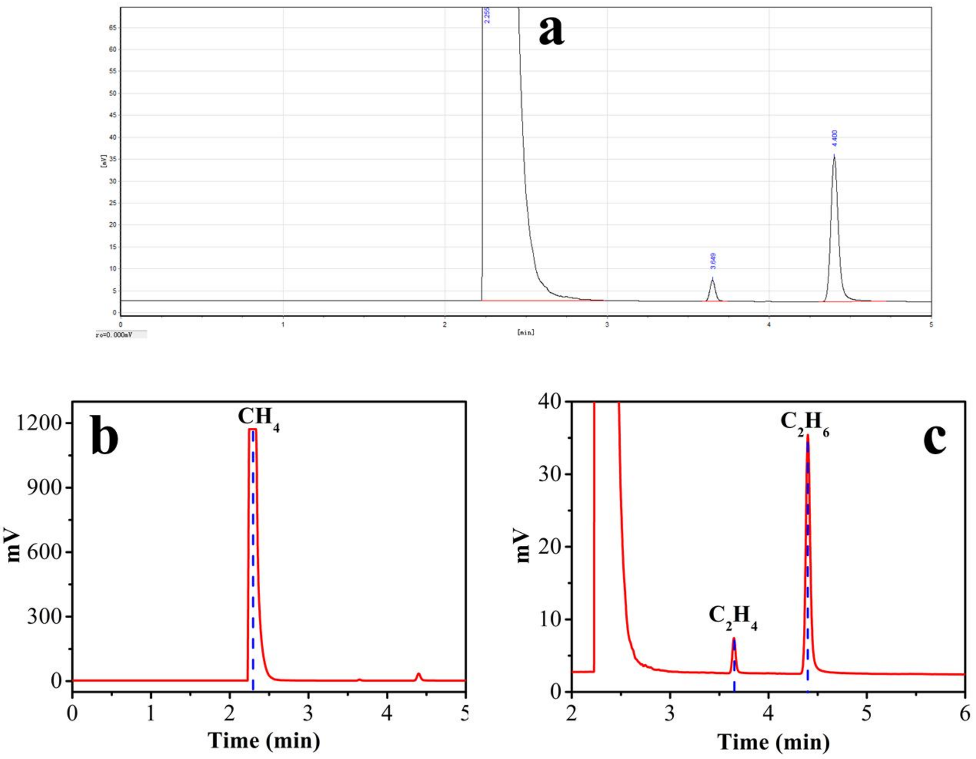

Supplementary Figure 12: Gas chromatography signals detected in the oxygen-free methane conversion experiment that was carried out over the 0.1-Ag-ZnO nanocatalyst in the flow mode with a gas flow rate of 10 mL/min. **a**, the original spectrum; **b**, the replot full spectrum; **c**, the replot enlarged spectrum. The calculated ratio of CH_4 conversion is 0.35%, the selection of C_2H_4 and C_2H_6 are 10.53% and 89.47%, respectively.

Supplementary Note 1: Turnover number calculations.

Reaction formula ： $CH_4 + 2O_2 \rightarrow CO_2 + 2H_2O$

The number of electrons gained and lost in the reaction: $8 \cdot e^{-1}$, we assume that all electrons were excited by light.

The amount of substance for 20 mL CH_4 : $n_1 = 20\times10^{-3}/22.4\ \mathit{mol} = 8.929\times10^{-4}\ \mathit{mol}$

The total amount of substance for the electron's gain and loss in the photocatalyst degradation of 20 mL CH_4 :

$$n_2 = 8\times8.929\times10^{-4}\ \mathit{mol} = 7.1432\times10^{-3}\ \mathit{mol}$$

For the 0.5 g 0.1-Ag composites:

The amount of substance for ZnO: $n_3 = 0.999\times0.5/81.39\ \mathit{mol} = 6.137\times10^{-3}\ \mathit{mol}$

The amount of substance for Ag: $n_4 = 0.001\times0.5/107.9\ \mathit{mol} = 4.6339\times10^{-6}\ \mathit{mol}$

For ZnO, the Turnover number: $n = 7.1432/6.137 = 1.164$

For Ag, the Turnover number: $n = 7.1432\times10^{3}/4.6339 = 1541.51$
